# Dynamic astrocytic complement C3 activation in the epileptic hippocampus

**DOI:** 10.3389/fneur.2025.1682488

**Published:** 2025-11-19

**Authors:** Mi Jiang, Alessia Romagnolo, Eleonora Aronica, Yu Wang

**Affiliations:** 1 Department of Neurology, The Third Xiangya Hospital, Central South University Xiangya Medical School, Changsha, China; 2 Department of Neurology, University of Michigan, Ann Arbor, MI, United States; 3 Department of (Neuro) Pathology, Amsterdam UMC, University of Amsterdam, Amsterdam Neuroscience, Amsterdam, Netherlands; 4 Ann Arbor VA Hospital, Ann Arbor, MI, United States; 5 Neuroscience Graduate Program, University of Michigan, Ann Arbor, MI, United States

**Keywords:** C3, temporal lobe epilepsy, status epilepticus, stratum lacunosum-moleculare, neuroinflammation

## Abstract

Complement C3 plays important roles in neuroinflammation and is significantly upregulated in hippocampal tissues from patients with mesial temporal lobe epilepsy (mTLE) that is characterized by neuronal loss and gliosis. However, the temporal and spatial expression of complement C3 in the epileptic brain remain unclear. In this study, we first confirmed the upregulation of C3 in resected hippocampal tissues encompassing the CA1–CA4 regions from patients with mTLE with hippocampal sclerosis (mTLE-HS) using RNA sequencing, and in an mTLE mouse model using microarray data from the GEO database. Using a novel floxed C3-IRES-Tdtomato knock-in mouse line, we identified sustained C3 overexpression mainly in reactive astrocytes within the hippocampal stratum lacunosum-moleculare. This activation emerged 3 days after pilocarpine-induced status epilepticus, progressively extended to the CA1 region by 3 weeks, and highlighted region- and stage-specific dysregulation of C3 during epileptogenesis.

## Introduction

Complement activation or dysregulation is increasingly recognized as a key contributor to neuroinflammatory and neurodegenerative diseases, including primary neurodegeneration such as Alzheimer’s disease (AD) (1–3) and amyotrophic lateral sclerosis (ALS) (4), as well as secondary neurodegeneration observed in epilepsy (5–7), multiple sclerosis (MS) (8, 9), and traumatic brain injury (10, 11). Complement is also implicated in neurodevelopmental and neuropsychiatric diseases, including schizophrenia (12, 13) and mood disorders (14), where overactivation of the complement cascade and increased microglia-mediated synaptic uptake disrupt brain maturation and circuit stability (15, 16). Among complement components, C3 serves as a central effector bridging innate immunity and neuroinflammation: upon activation, it is cleaved into C3a (17) and C3b (18, 19), which modulate astrocytic and microglial activity (19–22), as well as synaptic pruning (23). These effects of C3 on glial cells and synapses contribute to inflammatory and degenerative processes in the brain (20, 21, 23–26). The transcriptional profile of microglia in MS shows overlap with that observed in AD and ALS, suggesting that primary and secondary neurodegeneration may share common mechanisms (23, 27–29). Although it remains uncertain whether C3 activity represents a physiological process or a pathogenic response contributing to neural circuit remodeling and disease progression, such widespread circuit-level disturbances provide a conceptual framework to understand how C3-mediated complement activation might drive the maladaptive network remodeling underlying epileptogenesis. Supporting this framework, C3 is consistently upregulated in resected hippocampal tissues from patients with mesial temporal lobe epilepsy (mTLE) (5, 30) as well as in various experimental models (5, 31–33), highlighting its potential as a key mediator of neuroinflammatory network alterations.

Several studies suggest that C3 may contribute to epileptogenesis in mTLE via distinct mechanisms, some of which may be interrelated. First, C3 signaling via the C3a–C3a receptor (C3aR) axis promotes crosstalk between astrocytes and microglia, amplifying inflammatory cascades and neuronal injury (34); excessive C3a–C3aR activation can also drive microglial engulfment of inhibitory synapses, disrupting excitatory–inhibitory balance (22). Second, deposition of C3b on synaptic elements labels them for microglial engulfment, leading to aberrant synaptic pruning, pathological synapse loss, and may further imbalance of excitatory and inhibitory circuits (31, 35). Global genetic C3 knockout prevents status epilepticus (SE)–induced hippocampal neuronal loss and recognition memory deficits (36), supporting a causal role of C3 in seizure-related neurodegeneration (34). Conditional deletion of astrocyte-derived C3 or pharmacological inhibition of C3–C3aR signaling during the chronic phase suppresses seizures, protects inhibitory synapses from microglial engulfment, and restores inhibitory synaptic transmission in hippocampal neurons (22). When treatment is initiated in the early latent stage, it further alleviates epilepsy and cognitive impairment by attenuating C3-mediated inflammation and synaptic phagocytosis (37). Taken together, these findings indicate that C3 activation not only mediates neuroinflammatory responses but also contributes to maladaptive synaptic and circuit remodeling that underlies epileptogenesis, linking molecular, cellular, and network-level dysfunction to seizure-related pathology. Importantly, the effects of C3 modulation appear to be stage-dependent, with early latent-phase interventions generally more beneficial than prolonged or non-selective inhibition. Thus, precise targeting of C3 signaling may offer a promising strategy to attenuate neuroinflammation, preserve synaptic integrity, and suppress epileptogenesis in mTLE and related disorders.

Despite these findings, the spatiotemporal expression patterns of C3 during epileptogenesis remain poorly characterized, and it is unclear whether its expression follows distinct trajectories across different seizure stages. To address these questions, we utilized a novel floxed C3-IRES-Tdtomato knock-in (KI) mouse line to investigate C3 expression across different stages of epileptogenesis in the well-characterized mouse pilocarpine model of mTLE (38). This approach provides insights into the spatial, temporal, and cell type–specific regulation of C3 in epilepsy, informing potential strategies to modulate neuroinflammatory pathways in seizure disorders.

## Materials and methods

### Human and rodent sequencing data collection and analysis

Resected hippocampal tissues encompassing the CA1–CA4 regions were obtained from mTLE with hippocampal sclerosis (mTLE-HS) patients (*N* = 64) and age-matched controls (*N* = 13). All samples were collected with informed consent and approved by the institutional ethics committees of the participating medical centers (Science Committee of the BioBank and Medical Ethical Committee). Samples were obtained from the archives of the Departments of Neuropathology at Amsterdam UMC (Amsterdam, The Netherlands) and UMC Utrecht (Utrecht, The Netherlands), reviewed by two independent neuropathologists. Control tissue was postmortem from individuals without neurological disease. Clinical details are in the Supplementary data.

RNA sequencing and differential expression genes (DEGs) analysis followed established protocols (39). Gene expression differences between TLE-HS and controls were analyzed using edgeR and limma (40, 41), with genes meeting a BH-adjusted *p*-value < 0.05 considered differentially expressed. C3 expression was visualized in a boxplot.

For rodent mTLE, DEG analysis used the GSE73878 dataset from GEO, categorized into 7-, 28-, and 60-day following kainic acid (KA) administration. DEGs (*p*.adj < 0.05, |log2FC| > 1) were identified using GEO2R, with results displayed in volcano plots.

### Animals and pilocarpine injections

**Figure 1:**
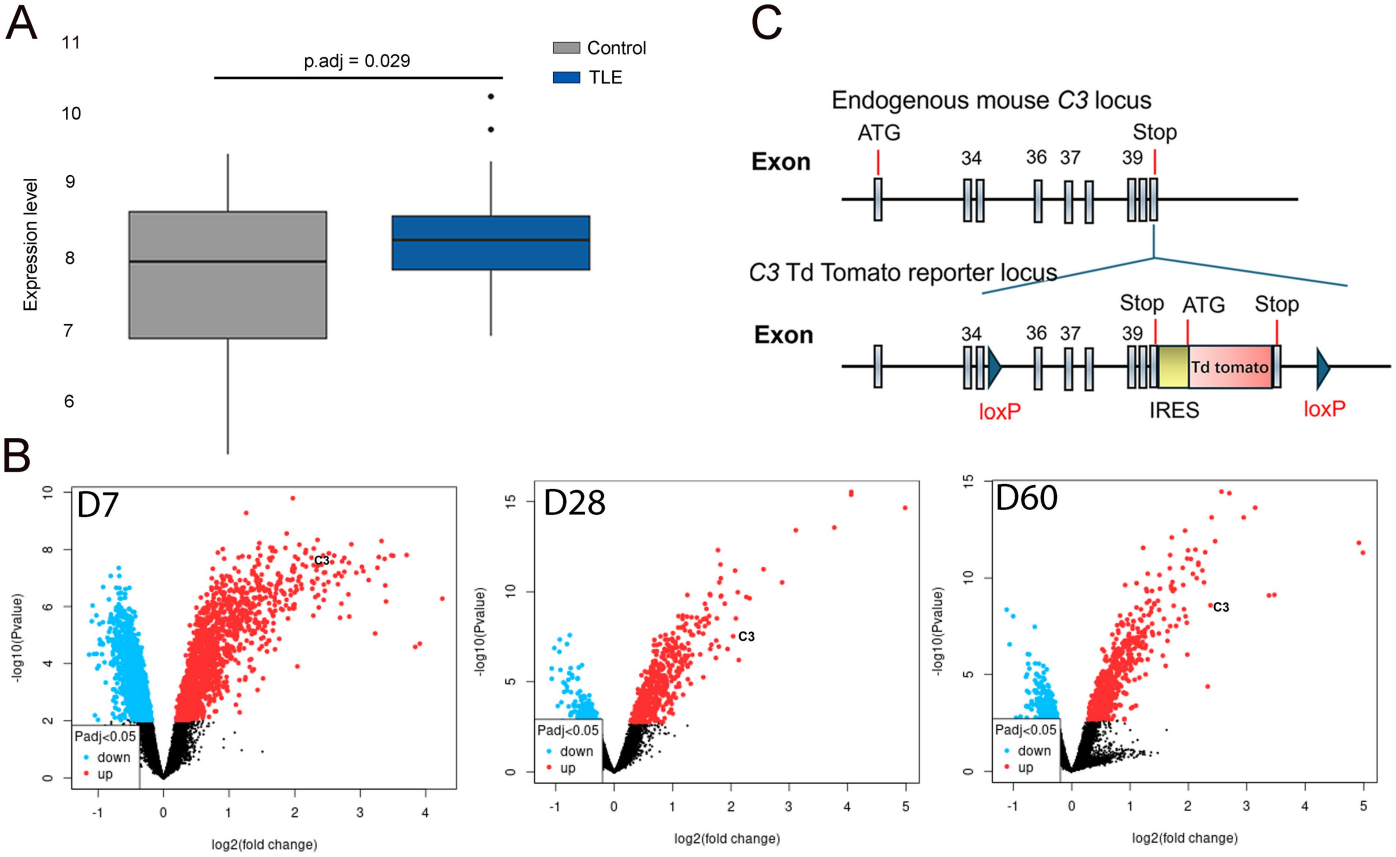
C3 expression is increased in human and rodent mTLE. **(A)** In the box plot, the horizontal axis represents the sample groups (control on the left and TLE-HS on the right), while the vertical axis shows the gene expression values. The black line within the box indicates the median gene expression value. The adjusted *p*-value (*p*.adj) of 0.029 is displayed at the upper center of the plot. The box plot demonstrates elevated C3 expression levels in the hippocampus of TLE-HS patients (blue box, right) compared to controls (gray box, left). **(B)** In the volcano plot, the *x*-axis represents the fold change (magnitude of expression difference), while the *y*-axis denotes statistical significance (expressed as the negative log10 *p*-value). The most statistically significant genes are located at the top (blue and red dots), while non-significant genes are clustered at the bottom (black dots). Significance is determined by a *p*.adj < 0.05, as indicated by the symbols in the bottom-left insets. The most upregulated genes appear on the right (red dots), while the most downregulated genes are on the left (blue dots). The left panel of the volcano plot shows C3 appearing in the upper right at 7 days post-induction. The middle panel shows C3 in the upper right at 28 days post-induction, and the right panel also shows C3 in the upper right at 60 days post-induction. These volcano plots indicate increased C3 expression in the hippocampus of KA-induced TLE at 7-, 28-, and 60-days post-induction. **(C)** Diagram of the floxed C3-IRES-TdTomato KI transgene. Schematic illustrating the endogenous mouse C3 locus and the C3-TdTomato reporter locus, which was created by inserting an IRES-TdTomato cassette after the endogenous stop codon, flanked by loxP sequences. This model enables both C3 reporter activity and the generation of conditional knockout animals.

Floxed C3-IRES-Tdtomato KI mice (C57BL/6 J) were obtained from Claudia Kemper at NIH (9), with the transgenic strategy shown Figure 1C. Genotyping was carried out by Transnetyx using probe and primer sets specific to the wild type C3 allele and the knocked-in C3-tdTomato allele. Both male and female mice were included in the experiments. Wilde-type (WT) male C57BL/6 J mice were obtained from Charles River Laboratories (Stock #027). Floxed C3-IRES-TdTomato KI mice and age-matched WT C57BL/6 J controls (6–8 weeks old) were housed under a 12-h light/dark cycle with ad libitum access to food and water. All procedures followed U.S. Public Health Service guidelines and were approved by the University of Michigan IACUC. To reduce peripheral cholinergic effects, mice received scopolamine (1 mg/kg, Sigma-Aldrich, S1875) intraperitoneally 30 min before pilocarpine (300 mg/kg, Sigma-Aldrich, P6503). SE in pilocarpine-treated mice was defined as ≥3 consecutive stage 4–5 convulsive seizures according to the Racine scale (42), characterized by forelimb clonus with rearing and falling, or a series of recurrent seizures without recovery of normal posture or responsiveness between episodes. In this study, SE was typically maintained for approximately 1 h before termination with diazepam (10 mg/kg, i.p.). Each group included 15 animals.

### Electroencephalogram (EEG) implantation

Animals were anesthetized with 4%–5% isoflurane and maintained at 1%–2% on a stereotaxic frame. The scalp was shaved and disinfected alternately with iodophor and saline. Ophthalmic ointment was applied to prevent eye dryness. After a midline incision, three burr holes were drilled, and screw electrodes (#8IE3639616XE; P1 Technologies) were implanted bilaterally and referenced to the cerebellum. Electrodes were connected to a 6-channel pedestal (#8K000229801F; P1 Technologies) and secured with dental acrylic for stable long-term EEG recording. Carprofen (#SML1713, Sigma-Aldrich) was administered preemptively and for 48 h postoperatively for analgesia.

### Video-electroencephalogram (vEEG) monitoring and analysis

Three to 5 days after electrode implantation, animals underwent continuous vEEG monitoring (Natus, Middleton, WI) for 1–3 weeks. During recordings, animals were tethered via a commutator and housed in custom-built cages allowing free movement. EEG and video were continuously acquired, and seizures and interictal epileptiform discharges (IEDs) were identified by manual review. IEDs were defined as transient, clearly distinguishable events from background activity, and seizures as repetitive, rhythmic discharges lasting longer than 10 s, as previously described (43).

### Immunohistochemistry

Following transcardial perfusion, brains were fixed in 4% paraformaldehyde overnight at 4 °C, sectioned (70 μm) using a Leica VT1000S vibratome, and processed for immunocytochemistry as free-floating sections. Primary antibodies used were Rabbit anti-RFP (1:500, Rockland #600–401–379), Mouse anti-Iba1 (1:500, Abcam #ab283319), and Mouse anti-GFAP (1:500, Sigma #G3893). Fluorescently conjugated secondary antibodies (1:500, Invitrogen, Alexa Fluor 488, 594, or 647) were applied, and nuclei were labeled with bisbenzimide (1:500, Invitrogen #H1398).

### Imaging acquisition and quantification

Images were acquired using Nikon and Leica5 confocal microscopes and analyzed with ImageJ (NIH, USA). Immunofluorescence images were acquired using a confocal microscope and analyzed in ImageJ. Regions of interest (ROIs) encompassing the entire hippocampus were manually delineated based on anatomical landmarks. For intensity analysis, mean fluorescence intensity within each ROI was measured, and background signal was subtracted using a nearby area devoid of specific staining. For density analysis, raw images were first converted to grayscale, thresholded to reduce background noise, and segmented. Positively stained cells or structures were identified using the “Analyze Particles” function with size and circularity filters. Immunostaining density was calculated as the total stained area per ROI (pixels^2^/ROI area), and C3^+^ density was expressed as the percentage of C3-immunopositive area relative to the total hippocampal ROI using a fixed fluorescence threshold in ImageJ. Data are reported as mean ± SEM (*N* = 3 independent animals per time point, *n* = 9 images per time point). For each animal, values from multiple sections were averaged and the animal mean was used for statistical analysis. Density = C3-positive area/ROI area; intensity = background-corrected mean fluorescence within the ROI.

For cell-type–specific C3 expression analysis, TdTomato-positive (C3-TdT^+^) cells were identified in the hippocampus using confocal microscopy. Sections were co-immunostained with GFAP (astrocyte marker) and Iba1 (microglial marker). Co-localization was assessed by manually counting C3-TdT^+^ cells that co-expressed GFAP or Iba1 within defined regions of interest (ROIs) using ImageJ. The percentage of co-localization was calculated as the number of double-positive cells (C3-TdT^+^/GFAP^+^ or C3-TdT^+^/Iba1^+^) divided by the total number of C3-TdT^+^ cells within the same ROI × 100. For each animal, three ROIs (*n* = 9 images in total) were analyzed and averaged; the mean value from each animal was used as one biological replicate (*N* = 3). Data were expressed as mean ± SEM.

## Results

### C3 is overexpressed in both human and mouse mTLE

In human RNA sequencing data, C3 expression was modestly but consistently elevated in the hippocampus of patients with TLE-HS compared to healthy controls (*p*.adj = 0.029), as shown in the box plot (Figure 1A). This mild upregulation suggests a potential involvement of complement activation in human epileptogenic tissue. In contrast, microarray analysis of the GEO dataset revealed a much stronger and time-dependent increase in C3 expression in the KA-induced mouse model of mTLE, with significant overexpression observed at 7, 28, and 60 days post-induction (Figure 1B). These findings indicate that C3 activation is not only present in human TLE-HS but is also dynamically regulated during epileptogenesis in experimental models, supporting a conserved role of complement signaling in the progression of epilepsy.

### C3-IRES-TdTomato KI mouse line develops consistent seizures following pilocarpine induction

**Figure 2:**
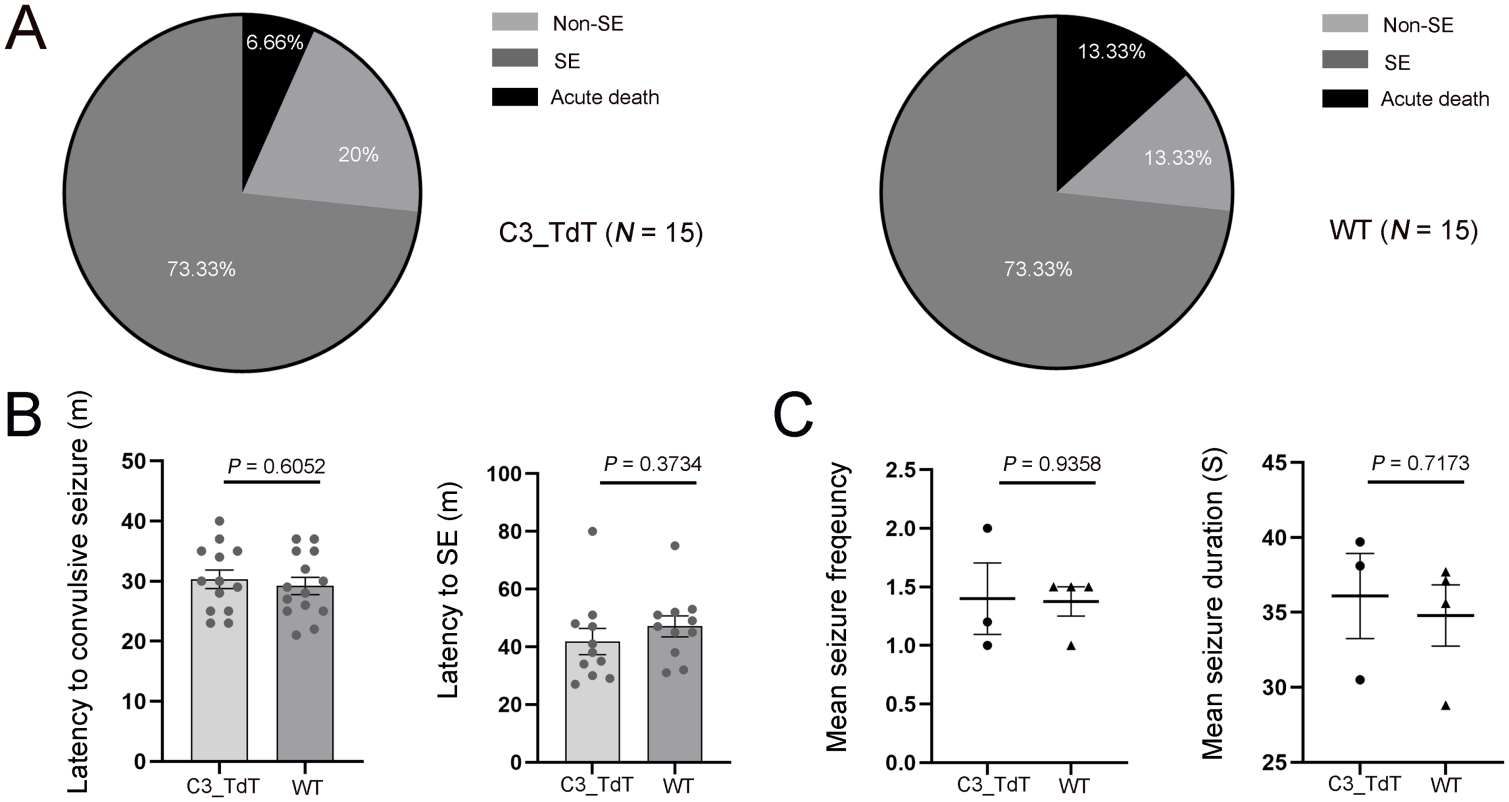
The C3-IRES-TdTomato KI mouse line develops seizures at a rate comparable to that of WT C57BL/6 J controls following pilocarpine induction. **(A)** The pie charts shows the outcomes of pilocarpine administration. Approximately 73.33% of pilocarpine-injected floxed C3-IRES-Tdtomato KI mice developed SE, 20% did not develop SE, and 6.66% experienced acute death following pilocarpine injection. A similar pattern was observed in wild-type C57BL/6 J control mice, with 73.33% developing SE, 13.33% not developing SE, and 13.33% experiencing acute death. Each group included 15 animals; **(B,C)** Data are presented as mean ± SEM. Each dot represents an individual mouse. Statistical comparisons were performed using an unpaired two-tailed *t*-tests, with statistical significance defined as *p* ≤ 0.05. Quantification of the latency from pilocarpine injection to the first convulsive seizure [30.31 ± 1.50 min for C3_TdT (*N* = 12) vs. 29.21 ± 1.42 min for WT (*N* = 12); *p* = 0.6052] **(B)**; Quantification of the latency from pilocarpine injection to SE onset [41.82 ± 4.52 min for C3_TdT (*N* = 10) vs. 47.09 ± 3.61 min for WT (*N* = 10); *p* = 0.3734]. Mean seizure frequency [1.40 ± 0.31 for C3_TdT (*N* = 3) vs. 1.38 ± 0.13 for WT (*N* = 4); *p* = 0.9358] and mean seizure duration (36.10 ± 2.84 s for C3_TdT vs. 34.80 ± 2.05 s for WT; *p* = 0.7173) **(C)**.

Observations from both patient mTLE hippocampal tissues and KA-induced mTLE motivated the use of C3-IRES-TdTomato KI mice to directly track C3 dynamics *in vivo*, allowing us to validate the findings under controlled experimental conditions. To validate the reporter model and evaluate whether the TdTomato insertion in C3-IRES-TdTomato KI mice affects seizure development, we compared SE induction rate, mortality, latency to the first convulsive seizure, and SE onset between C3-IRES-TdTomato KI mice (*N* = 15) and WT C57BL/6 J mice (*N* = 15). Following pilocarpine injection, both groups showed comparable SE induction rates, non-response rates, and acute mortality (Figure 2A). The latency to the first convulsive seizure (*p* = 0.6052) and the average latency (*p* = 0.3734) from pilocarpine administration to SE onset were also similar between the two groups (Figure 2B). C3-IRES-TdTomato KI mice (*N* = 3) developed spontaneous seizures within 2 weeks of acute SE, and no significant differences in seizure frequency (*p* = 0.9358) or duration (*p* = 0.7173) were observed between groups (Figure 2C). Due to limited video resolution, the severity of individual seizures could not be precisely assessed. Nevertheless, these findings suggest that the C3_TdT transgene does not substantially affect seizure susceptibility or the course of epileptogenesis.

### Spatiotemporal dynamics of C3 in C3-IRES-TdTomato KI mouse epileptic brains

**Figure 3:**
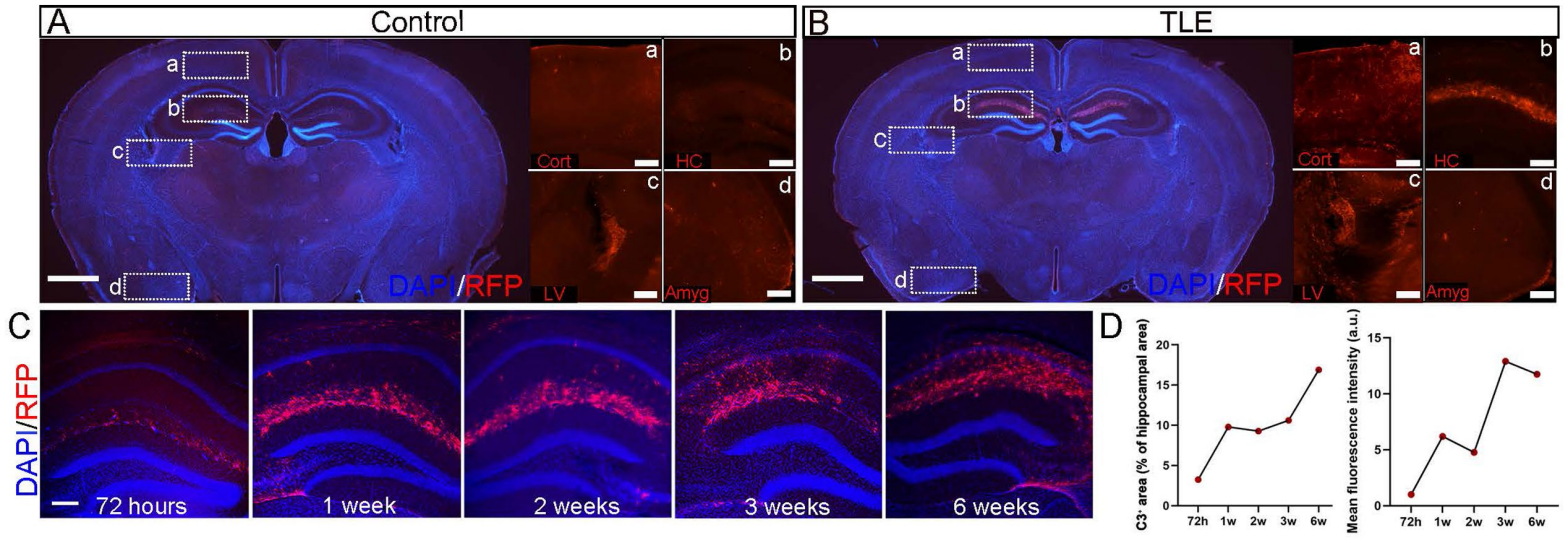
Temporal profile of hippocampal C3 activation after SE. **(A,B)** Immunostaining of whole-brain sections from C3-IRES-tdTomato KI mice in the pilocarpine-injected non-SE control group **(A)** and SE-induced TLE group **(B)**. In each group, representative merged images of DAPI (blue) and RFP (red) in whole-brain sections are shown (scale bar: 1,000 μm), along with enlarged views of RFP (red) signals in the cortex (A), hippocampus (B), lateral ventricle (C), and amygdala (D) (scale bar: 200 μm). Cort, cortex; LV, lateral ventricle; HC, hippocampus; Amyg, amygdala. **(C)** Immunostaining of whole-brain sections from C3-IRES-tdTomato KI mice in the pilocarpine-induced SE group. Shown are representative merged images of DAPI (blue) and RFP (red) immunostaining in whole-brain sections highlighting the hippocampal region at 72 h, 1 week, 2 weeks, 3 weeks, and 6 weeks after SE induction. Scale bar: 200 μm. **(D)** Quantitative analysis of C3^+^ area (% of hippocampal area) and mean fluorescence intensity in the hippocampus (a.u.) at 72 h, 1 week, 2 weeks, 3 weeks, and 6 weeks after SE induction revealed a progressive increase in C3^+^ area over time, accompanied by a slight decrease in mean intensity at 6 weeks. Data are presented as mean ± SEM (*N* = 3 independent animals per time point, *n* = 9 images per time point). Each point represents the mean value for one time group; lines connect means to illustrate the temporal trend. No statistical analysis was performed; data are shown to indicate temporal trends. Arbitrary unit (a.u.).

Having established the validity of this reporter line, we next applied it to a pilocarpine-induced TLE model, which revealed robust upregulation of C3 expression during epileptogenesis. In pilocarpine-injected no SE control group, TdTomato signals were confined to the choroid plexus within the subarachnoid space, with no detectable expression in the cortex, hippocampus, or amygdala (Figures 3Aa–d). In SE-induced TLE group, intense TdTomato signals were significantly increased in the stratum lacunosum-moleculare (SLM) layer of the hippocampus, with minimal expression in the choroid plexus and cortex, and no detectable expression in the amygdala (Figures 3Ba–d).

To investigate the dynamic expression of C3 expression in epileptic brains, we performed a time-series immunohistochemical staining of TdTomato in C3-IRES-Tdtomato KI mice brains following pilocarpine-induced SE. Interestingly, no detectable TdTomato expression was observed in the hippocampal formation within 24 h in SE group (data not shown), suggesting that C3 overexpression is not a response to acute seizures. At 72 h after SE induction (acute phase), TdTomato fluorescence was weak and sparsely distributed within the SLM of the hippocampus. At 1 and 2 weeks post-SE (latent phase), both the density and fluorescence intensity of TdTomato^+^ cells progressively increased and began to extend into the CA1 region. By 3 and 6 weeks post-SE (chronic phase), TdTomato labeling became prominent throughout CA1, although the mean fluorescence intensity showed a slight decline at 6 weeks (Figures 3C,D). This temporal and spatial progression of C3 expression parallels the transition from the latent to the chronic epileptic phase, during which spontaneous recurrent seizures typically emerge.

### Cell type sources of C3 in C3-IRES-TdTomato KI mouse mTLE

**Figure 4:**
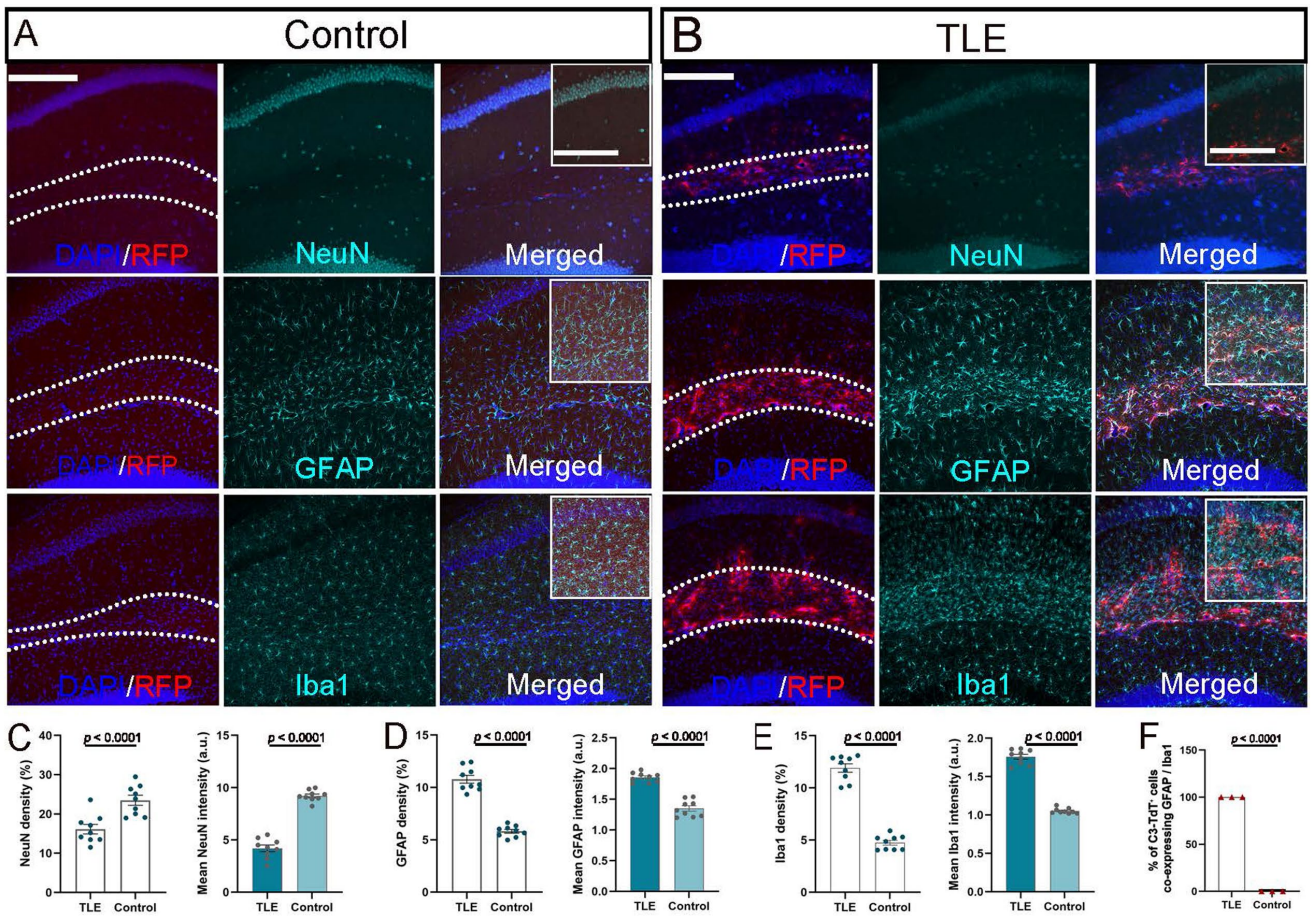
Cellular source of C3 expression in epileptic hippocampus. **(A,B)** Immunostaining of hippocampal sections from C3-IRES-tdTomato KI mice 2 weeks after pilocarpine injection in the non-SE control group **(A)** and SE-induced TLE group **(B)**. Representative merged images show DAPI (blue) and RFP (red) signals highlighting the hippocampal SLM layer (outlined by dashed lines; scale bar: 200 μm). Corresponding immunostaining for NeuN, GFAP, and Iba1 (green) is shown in the middle column, with fully merged images presented in the right column. Insets represent higher-magnification views of the SLM region (boxed area in merged images; inset scale bar: 100 μm). (**C–F)** Quantitative analysis of NeuN^+^ cell density and mean fluorescence intensity in the CA1 area **(C)**, and of GFAP^+^ **(D)** and Iba1^+^ **(E)** immunoreactivity in the SLM region, showing significant differences between groups (*p* ≤ 0.0001). Quantification of the percentage of C3-TdT^+^ cells co-expressing GFAP or Iba1 (*p* ≤ 0.0001) **(F)**. Data are presented as mean ± SEM (*N* = 3 independent animals per group, *n* = 9 images per group). For panels **C–E**, each point representing one image. For panel **F**, each point representing one animal. Statistical significance was determined using unpaired two-tailed *t*-tests. Arbitrary unit (a.u.).

NeuN staining revealed a significant neuronal loss in the CA1 region of the SE-induced TLE group compared with the pilocarpine-injected non-SE control group, as evidenced by the reduced neuronal density and fluorescence intensity (*p* ≤ 0.0001) (Figures 4A–C). Quantitative analysis revealed that the hippocampus, particularly within the SLM, in the TLE group 2 weeks after pilocarpine-induced SE showed a marked and statistically significant (*p* ≤ 0.0001) increase in both astrocytosis and microgliosis, as reflected by the higher density and mean fluorescence intensity of GFAP^+^ astrocytes and Iba1^+^ microglia compared with the non-SE group (Figures 4A–E). All C3-TdT^+^ cells co-expressed GFAP^+^ astrocytes, confirming astrocytes as the primary cellular source of C3 in TLE, while the absence of TdTomato staining in Iba1^+^ microglia indicates that microglia do not contribute to C3 expression in the epileptic SLM region (*p* ≤ 0.0001; Figure 4F). Astrocytes appear to be the major source of C3 expression in the epileptic hippocampus, accompanied by neuronal loss and gliosis in the epileptic hippocampus.

## Discussion

In this study, we found a modest yet statistically significant elevation of C3 expression in resected hippocampal tissues from patients with TLE-HS compared with controls, aligning with previous observations (5, 30) and supporting a role for C3 activation in human TLE. These findings were further supported by analyses of sequencing data from KA-induced mTLE models retrieved from GEO datasets. The more pronounced changes observed in KA-induced mTLE model may reflect sampling at earlier disease stages compared with chronic patient tissues obtained after years of recurrent seizures. To directly track complement activation dynamics under controlled conditions, we employed C3-IRES-TdTomato KI mice. Validation of the C3-IRES-TdTomato KI model confirms its reliability for monitoring complement activation during epileptogenesis. The comparable seizure parameters between KI and WT mice indicate that TdTomato insertion does not interfere with endogenous C3 function or seizure susceptibility. This ensures that the observed C3 dynamics truly reflect physiological complement responses, providing a robust *in vivo* tool to investigate the spatiotemporal regulation of C3 and its role in disease process. The upregulation of C3 expression was further validated in a pilocarpine-induced TLE model using C3-IRES-TdTomato KI mice, which revealed robust and dynamic C3 upregulation during epileptogenesis. Although the gene-expression data were derived from a KA-induced model and histological analyses were performed in the pilocarpine model, which differ in their induction mechanisms and certain neuropathological features, the consistent upregulation of C3 across both models supports the general relevance of complement dysregulation in mTLE, despite potential limitations in direct cross-model comparability.

C3 expression exhibited a distinct spatiotemporal progression following pilocarpine-induced SE. It first appeared in the SLM around day 3, corresponding to the early latent phase, and gradually extended toward the CA1 pyramidal layer by 1–2 weeks, becoming markedly elevated in CA1 during the chronic phase at 3–6 weeks post-SE. The SLM, where distal dendrites of CA1 pyramidal neurons receive excitatory inputs from entorhinal layer III via the perforant path, also integrates convergent inputs from two major excitatory pathways originating in the entorhinal cortex (EC), namely the temporoammonic and alvear pathways (44, 45), as well as modulatory inputs from somatostatin-positive inhibitory OLM interneurons (46). Previous study showed that both EC layer III neurons and CA1 pyramidal neurons are particularly vulnerable in the mTLE mouse model (47), and increased C3 expression has been detected in the EC of mTLE patients (30). Such anatomical and pathological convergence highlights the SLM as a critical hub for information integration and modulation within CA1. C3-mediated complement activation in this region may disrupt cortical–hippocampal communication and alter the local excitation–inhibition balance along the EC–CA1 pathway, key processes underlying epileptogenesis and disease progression. Consistent with the regional specificity observed, our results indicate that astrocytes are the mainly cellular source of C3 in the SLM, a region showing pronounced gliosis and concurrent microglial activation (as indicated by GFAP and Iba1 expression, respectively) in the epileptic hippocampus. All C3-tdTomato-positive cells co-localized with GFAP, whereas no signal was detected in Iba1-positive microglia, indicating that C3 expression is largely confined to astrocytes. The initial SLM-restricted increase, accompanied by astrocyte and microglia activation, likely reflects the dynamic interplay between C3, astrocytes, and microglia during the latent phase. The subsequent spread to CA1 suggests secondary propagation of gliosis and complement-mediated synaptic remodeling, marking a transition from a localized astrocytic response at the entorhinal–hippocampal input zone to broader recruitment of hippocampal circuits during chronic epileptogenesis. This astrocyte-specific complement activation aligns with prior evidence that glial C3 upregulation contributes to neuroinflammation and synaptic remodeling in epilepsy (34, 48). As astrocytes are central regulators of the neuroimmune microenvironment (49), elevated astroglial C3 expression may represent a key mechanism linking glial reactivity to complement-mediated synaptic dysfunction and network hyperexcitability in TLE.

Recent studies suggest that hyperactive inhibitory neurons in the CA1 region can activate microglia through GABA signaling, leading to selective phagocytosis of C3-tagged inhibitory synapses and disruption of the excitation–inhibition balance during the chronic stage (around 3 weeks after KA induction) (22). This mechanism may account for the progressive expansion of C3 expression observed in our model, from initial elevation in the SLM to later involvement of CA1. In the hippocampus, GABAergic signaling is strong around the somatic layer in CA1 (50), whereas the SLM provides more modulatory, dendrite-targeted inhibition primarily mediated by somatostatin-positive OLM interneurons (51). The temporal and spatial pattern of C3 upregulation observed in this study coincides with the reported window of microglia-mediated pruning of inhibitory synapses. Excessive GABA release from hyperactive interneurons in the SLM and CA1 may trigger microglial activation and local complement engagement, while astrocytic C3a–C3aR signaling could further amplify these processes across hippocampal layers. Such a feed-forward loop may gradually transform focal hyperactivity into widespread hippocampal network remodeling during epileptogenesis.

In this study, we employed a novel floxed C3-IRES-TdTomato KI mouse line, which provides a powerful and versatile tool to investigate the causal roles of complement signaling in epileptogenesis and associated comorbidities. This model faithfully reports endogenous C3 expression, enabling precise tracking of its spatiotemporal dynamics and cellular sources *in vivo*. Moreover, when crossed with cell type– or stage-specific Cre-driver lines, it permits conditional knockout or rescue of C3, facilitating mechanistic dissection of astrocytic versus microglial contributions. In combination with chemogenetic or optogenetic approaches, this system further allows direct evaluation of how complement activation influences network excitability, glial signaling, and seizure susceptibility. Together, these capabilities make the C3-IRES-TdTomato KI mouse a robust experimental platform for probing how selective genetic or pharmacological modulation of complement signaling affects epileptogenesis, neuroinflammation, and long-term circuit reorganization. Collectively, our findings and this experimental framework lay the foundation for future studies to elucidate how astrocyte-derived C3 orchestrates maladaptive neuron–glia interactions and whether targeting specific C3 signaling branches can mitigate pathological remodeling in temporal lobe epilepsy.

This study has several limitations. First, due to the lack of detailed clinical information (e.g., etiological subtypes of TLE), we were unable to determine whether distinct epilepsy syndromes are associated with differential C3 expression levels. Second, although we demonstrated that C3 is predominantly expressed by astrocytes and localized to the SLM in the mouse model of TLE, these spatial and cell type–specific findings have not yet been validated in human tissue, which limits their translational interpretation. Third, both human and animal RNA sequencing datasets represent bulk-tissue analyses, lacking cell type–specific resolution and quantitative validation. Finally, because longitudinal EEG monitoring was not performed, we could not directly correlate C3 expression dynamics with seizure onset, frequency, or severity.

Future work should elucidate the causal and mechanistic roles of astrocyte-derived C3 in TLE and validate C3 dysregulation through complementary molecular approaches. A key question is whether C3 upregulation in the hippocampal SLM represents an initiating event in epileptogenesis or a secondary response to neuronal injury. Defining the cellular sources and upstream regulators of C3, including possible inputs from microglia and endothelial cells, will be essential. Mechanistically, clarifying how C3a–C3aR–mediated astrocyte–microglia signaling and C3b-dependent synaptic pruning influence the excitatory–inhibitory balance and circuit remodeling within the SLM–CA1–EC pathway will advance our understanding of complement-driven network pathology. Integrating time-resolved molecular profiling with continuous EEG monitoring could further reveal how dynamic complement activation correlates with seizure progression. To address these questions, future studies should combine C3 inhibitors, conditional knockout or rescue approaches, and targeted pathway analyses to define the specific contributions of distinct C3 signaling branches and assess whether selective modulation of C3 can prevent or reverse maladaptive circuit reorganization during epileptogenesis.

## Data Availability

The original contributions presented in the study are included in the article/Supplementary material, further inquiries can be directed to the corresponding author.
